# Characteristics of Biohydrogen Production and Performance of Hydrogen-Producing Acetogen by Increasing Normal Molasses Wastewater Proportion in Anaerobic Baffled Reactor

**DOI:** 10.1155/2020/8885662

**Published:** 2020-06-05

**Authors:** Xuejia Gu, Yufeng Wang, Huaibo Li, Ji Li, Shuo Wang

**Affiliations:** ^1^Institute of Soil Fertilizer and Environment Resources, Heilongjiang Academy of Agricultural Sciences, Harbin 150086, China; ^2^Key Laboratory of Agricultural Environment of Northeast Plain, Ministry of Agriculture and Rural Affairs, Harbin 150086, China; ^3^Jiangsu Key Laboratory of Anaerobic Biotechnology, School of Environment and Civil Engineering, Jiangnan University, Wuxi 214122, China; ^4^Jiangsu College of Water Treatment Technology and Material Collaborative Innovation Center, Suzhou 215009, China; ^5^Department of Civil Engineering, Schulich School of Engineering, University of Calgary, Calgary, Canada T2N 1N4

## Abstract

The biohydrogen production efficiency and performance of hydrogen-producing acetogen in a four-compartment anaerobic baffled reactor (ABR) were studied by gradually increasing the influent normal molasses wastewater (NMWW) proportion. When the influent NMWW proportion increased to 55%, ABR could develop microbial community with methanogenic function in 63 days and reach a stable operation. When the influent NMWW proportion increased to 80% and reached a stable state, ethanol fermentation was established from butyric acid fermentation in the first three compartments, whereas butyric acid fermentation in the fourth compartment was strengthened. The average biohydrogen production yield and biohydrogen production capacity by COD removal increased to as high as 12.85 L/day and 360.22 L/kg COD when the influent NMWW proportion increased from 55% to 80%, respectively. Although the biogas yield and the specific biogas production rate reached 61.54 L/day and 232 L/kg MLVSS·day, the biohydrogen production yield and specific biohydrogen production rate were only 12.85 L/day and 48 L/kg MLVSS·day, which results in hydrogen consumption by homoacetogenesis and methanogenesis.

## 1. Introduction

A considerable amount of high-strength organic wastewater has been discharged into aquatic systems with poor treatment performance, which causes serious water pollution and destroys the ecological environment and poses a threat to the environment and human health [1]. Given the increasing demand for eliminating organic wastewater pollution, the ultimate goal is to achieve waste minimization and clean manufacturing [2]. Biohydrogen production by fermentation can utilize hydrogen-producing microorganisms to metabolize organic matters to produce hydrogen in anaerobic conditions and acidic circumstance [3]. At present, the basic principle of biohydrogen production is mainly based on the acidogenic fermentation of hydrogen-producing bacteria [4]. The terminal liquid products of fermentation are ethanol, acetic acid, propionic acid, and butyric acid [5]. Given the limited degradation of soluble products, most hydrogen is not released in the form of H_2_, which considerably limits the hydrogen conversion rate and becomes a technical bottleneck restricting the development and application of the biological hydrogen production technology through fermentation [6–8].

Normal molasses wastewater (NMWW) is an important byproduct in beet sugar and sugar cane factories [9], in which a large amount of molasses wastewater containing high-strength chemical oxygen demand (COD) in the range of 80000–130000 mg/L is produced and gradually becomes one of the most polluted wastewater in the food industry. However, NMWW is an excellent substrate for fermentative biohydrogen production because it contains a considerable amount of sucrose, glucose, fructose, nitrogen, and vitamins [10]. Upflow anaerobic sludge blanket reactor [11], which can recover energy in the form of methane through the NMWW treatment process, has been utilized for NMWW treatment. Nevertheless, with high organic-loading wastewater, high biogas flux often leads to biomass loss [12].

The principle of anaerobic baffled reactor (ABR) is to set up a series of vertical baffles in the reactor; thus, the wastewater can be introduced up and down along the baffle in ABR and then pass through the sludge bed of each compartment [13]. The operation flow path of ABR is similar to the plug flow process, which presents the provision of upper and lower baffles to form a compartment [14]. Such process can connect and effectively separate microorganisms from methanogenic and acidogenic phases [15]; therefore, two-phase fermentation can be established in a single reactor. Suaisom et al. [16] found that the increase in organic-loading rate eliminates methanogens and subsequently decreases methane production. Nachaiyasit and Stuckey [17] studied the effect of low temperature on the performance of ABR and discovered that the temperature had no significant effect on COD removal under medium organic-loading conditions. However, when the temperature was further reduced to 15°C, the performance of ABR decreased significantly; the possible reason could be due to the inhibition of bioactivity and the remarkable increase of half saturation degradation constant of volatile fatty acids (VFAs).

The present study aims at enhancing the treatment efficiency of NMWW from a macroscopic perspective by utilizing the characteristics of biological phase separation in enhancing the microbial activity of hydrogen-producing acetogen. Based on the good separation of the microbial community in ABR, this study intends to explore the combination of the fermentation bacteria and hydrogen-producing acetogen through the start-up of ABR. The biohydrogen production method on the basis of increasing the NMWW proportion (increasing COD) was performed to provide substantial biohydrogen production from NMWW.

## 2. Materials and Methods

### 2.1. NMWW and Inoculation Sludge

The NMWW, with an initial pH ranging from 5.3 to 5.8, was obtained from a sugar beet factory, and small amounts of urea and K_2_HPO_4_ were added to regulate the COD/N/P ratio to 200–500 : 5 : 1. The inoculated aerobic activated sludge was obtained from the local Wastewater Treatment Plant (Harbin, China), and anaerobic activated sludge (AnAS) was collected in a beer brewhouse. The AnAS was initially filtered and washed to remove inorganic particles. The first and fourth compartments were inoculated with aerobic and anaerobic sludge, respectively. Compartments 2 and 3 were inoculated with mixed aerobic activated sludge and AnAS in the proportions of 2.5 : 1 and 1 : 2.5 (*v*/*v*), respectively, and the initial mixed liquor volatile suspended solids (MLVSS) in each compartment were 6.9, 14.8, 23.1, and 22.7 g/L.

### 2.2. ABR Set-Up

**Figure 1:**
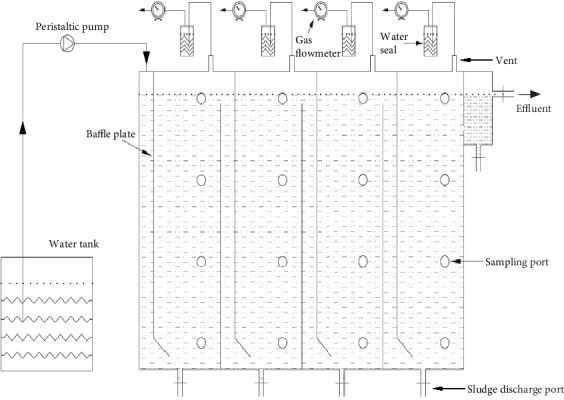
Schematic of ABR.

A four-compartment ABR with a total volume of 28.75 L was adopted in the experiment (Figure 1). Each compartment had equal size, with downflow and upflow chambers of 2.5 cm and 11.5 cm in width, respectively. NMWW was introduced to the bottom of the upflow chamber and finally drained through a guide plate with an inclination of 45° at the downflow chamber. The sampling ports were arranged at different heights of each compartment, and an effluent pipe was set at the top to connect with the water seal. The sealed water bottle and wet gas flowmeter were filled with HCl (pH of 3) to prevent gas dissolution, and the ABR temperature was maintained at 35 ± 1°C. The hydraulic retention time was 24 h in the ABR, and the initial influent NMWW proportion was 30%.  Table 1 lists the start-up stages of the ABR and the main operational parameters.

### 2.3. Analytical Methods

COD, pH, alkalinity (ALK, in terms of CaCO_3_), and mixed liquor volatile suspended solids (MLVSS) were regularly determined by the standard method (APHA [18]). The morphology of AnAS was observed by a scanning electrical microscope (JSM-5610). Biogas production in each compartment was measured by a wet gas flow meter, and the fermentation biogas, VFA, and ethanol compositions and contents were measured by gas chromatography (GC-122 and 4890G) [8].

## 3. Results

### 3.1. AnAS Biomass and Morphology

**Figure 2:**
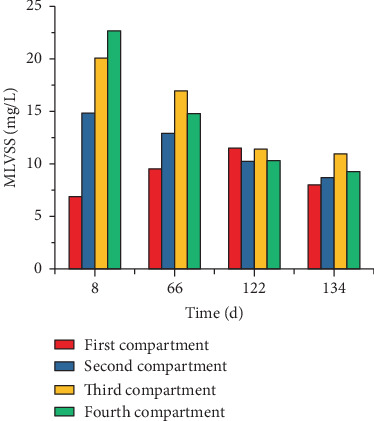
Biomass in each ABR compartment.

The biomass in each compartment presented a downward trend and reached the lowest values on day 62, which were the MLVSS values of 9.5, 12.9, 17.0, and 14.8 g/L, respectively (Figure 2). Then, the biomass gradually increased and remained stable along with the operation of ABR. In Stage II, the ABR system reached a steady state on day 101, and the respective biomass in each compartment stabilized at 13.5, 11.0, 12.5, and 10.4 g/L. With the increase in the NMWW proportion to 80% in Stage III, the operation of ABR achieved a stable state on day 127, and the MLVSS decreased to 8.0, 8.5, 12.1, and 9.9 g/L, respectively.

With the increase in the NMWW proportion in Stage I, the apparent characteristics of AnAS in each compartment did not change. However, the internal morphology changed significantly in each compartment.

**Figure 3:**
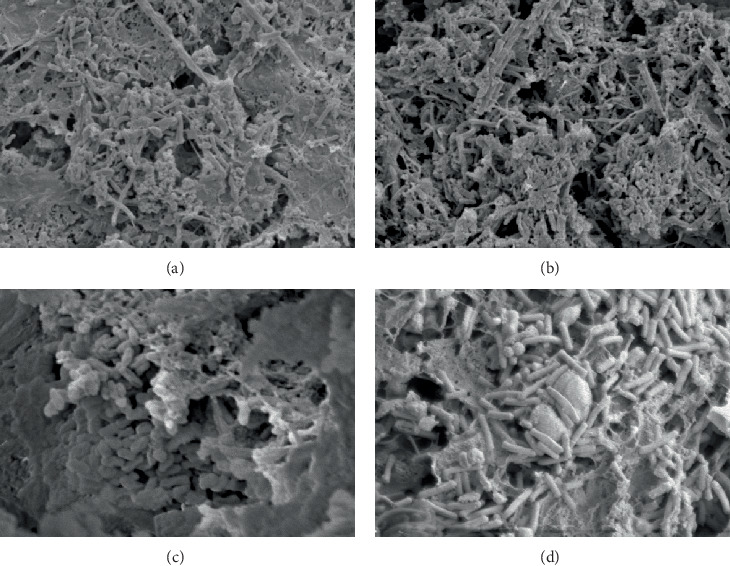
AnAS morphology in Stage I ((a) compartment 1, 5 k; (b) compartment 2, 5 k; (c) compartment 3, 8 k; and (d) compartment 4, 10 k).

As shown in Figure 3, the AnAS in compartment 1 was attached to the filament, and the microorganisms were mainly composed of *bacilli* and *cocci*. The dominant species in compartment 2 were mainly *bacilli*, and large numbers of filamentous bacteria were observed. *Bacilli* in compartment 3 were prevalent, and a large number of *Brevibacterium* and few *cocci* were observed in compartment 4.

**Figure 4:**
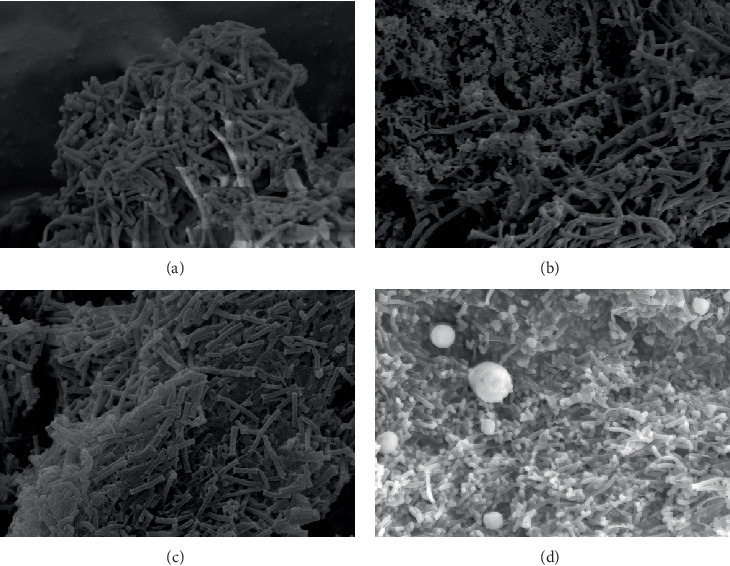
AnAS morphology in Stage II ((a) compartment 1, 5 k; (b) compartment 2, 5 k; (c) compartment 3, 5 k; (d) compartment 4, 5 k).

As shown in Figure 4, the morphology of microorganisms in the four compartments changed in Stage II, the *cocci* in compartment 1 gradually disappeared, and long *bacilli* become prevalent. In comparison with Stage I, the *Brevibacterium* in compartment 2 decreased. The dominant bacteria in compartment 3 was still *bacilli*, and the quantity of long bacilli increased. The AnAS in compartment 4 consisted of *bacilli*, and the number of *cocci* remarkably increased.

**Figure 5:**
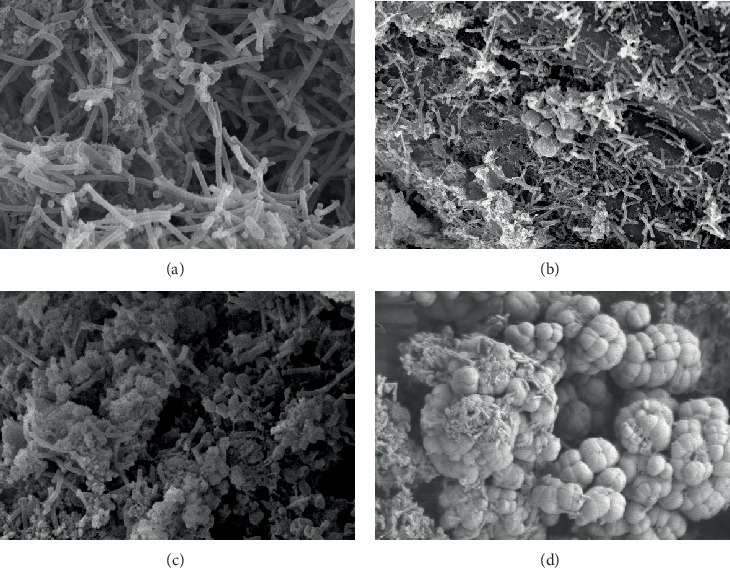
AnAS morphology in Stage III ((a) compartment 1, 6 k; (b) compartment 2, 3 k; (c) compartment 3, 5 k; (d) compartment 4, 4 k).

As depicted in Figure 5, the morphology of the microorganisms in the four compartments significantly changed with the increase in the NMWW proportion to 80%. The main microbial community in compartment I was *bacilli*, and the species became abundant. In comparison with Stages I and II, *cocci* began to appear in compartment 2. The short *bacilli* in compartment 3 gradually disappeared, whereas the *cocci* increased with the ABR operation. Additional *cocci* were observed in compartment 4, and the number of plump cells significantly decreased. This result could be due to the depletion of organic substances in compartments 1–3. The lack of substances finally led to a decline in microbial activity. The discrepancy of pH and alkalinity in each compartment also provided the foundation for the formation of the stable microbial community.

### 3.2. Biogas Production

**Figure 6:**
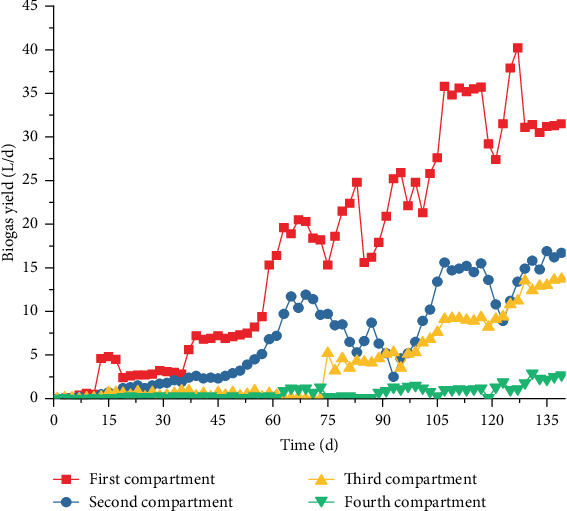
Biogas production in each compartment.

**Figure 7:**
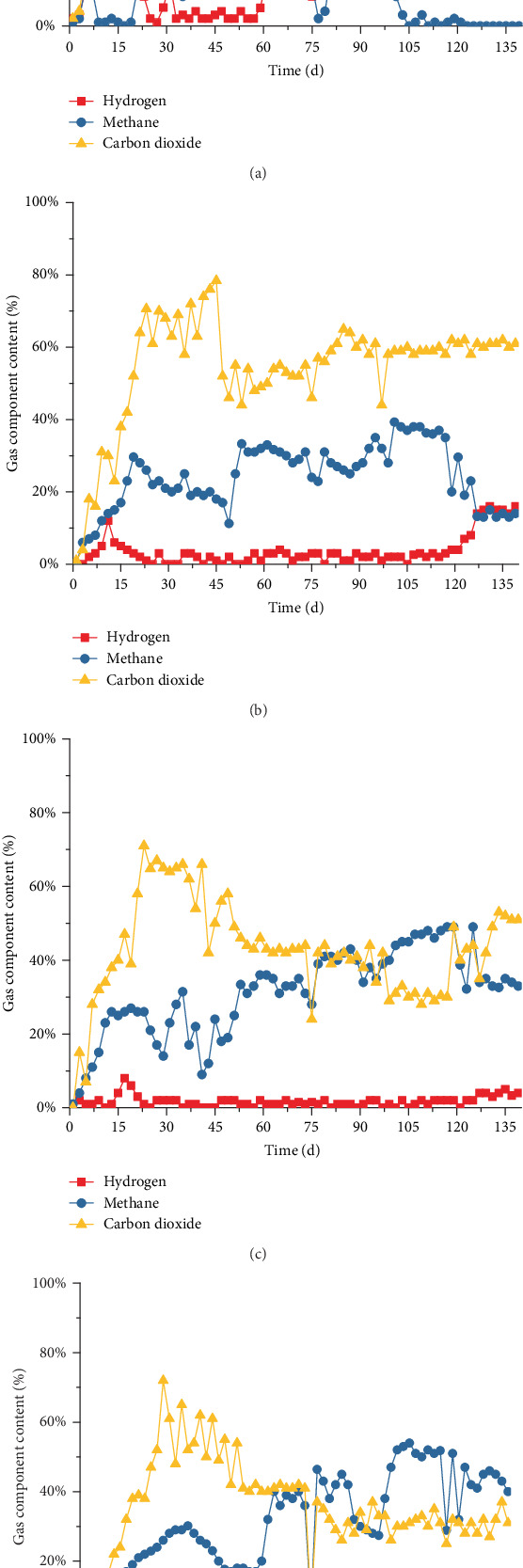
Variation in biogas concentration ((a) compartment 1; (b) compartment 2; (c) compartment 3; and (d) compartment 4).

With the start-up and operation of ABR, the biogas fluctuation was discovered in each compartment in the first 40 days. After 50 days of operation, the biogas production rate showed a steady upward trend and remained stable from days 64 to 73. Given the influence NMWW proportion of 55% and 80%, the biogas production rate fluctuated at the initial period of Stages II and III, and the stable state of operation was restored on days 108–117 (Stage II) and days 130–139 (Stage III). During the stabilization period of the three stages, the biogas production rates in compartment 1 were 19.32, 35.43, and 31.16 L/day (Figure 6), and the hydrogen contents were maintained at 13.6%, 27.1%, and 31.9%, respectively (Figure 7). The maximum biogas production rate in compartment 1 occurred in Stage II but decreased in Stage III, and the biogas production rates in compartments 2–4 showed a gradual increase as the NMWW proportion increased. From Stages I to III, the biogas production rates in compartment 2 were 10.79, 15.07, and 15.88 L/day, and the hydrogen contents were 2.5%, 2.6%, and 15%, respectively. The biogas production rates in compartment 3 were 0.08, 9.12, and 13.18 L/day, and the hydrogen contents were 1.4%, 0.9%, and 0.7% in compartment 4, respectively.

In Stage I, the average CH_4_ contents in each compartment were 9.9%, 30.1%, 33.2%, and 38.2% (Figure 7), which showed an increasing trend along the process, whereas the corresponding CO_2_ content showed a decreasing trend. However, with the increase in the NMWW proportion, the variations in CH_4_ and CO_2_ contents in compartment 1 showed different properties to other compartments. During Stage II, the CH_4_ content in compartment 1 significantly reduced to 0.9%, and a small amount of CH_4_ was detected in Stage III. Thus, the methanogenic activity of AnAS was completely inhibited. The methanogenic activity of AnAS in compartments 2–4 was enhanced in Stage II. The CH_4_ content in compartments 2–4 increased to 36.7%, 47.5%, and 51.7%, with CO_2_ contents of 58.8%, 29.9%, and 32.0%, respectively (Figure 7). In Stage III (COD of 8000 mg/L), the methanogenic activity of AnAS in compartments 2–4 was inhibited with different degrees. The methane proportion in compartments 2–4 decreased to 13.6%, 33.8%, and 43.1%, respectively, which may be due to the increase in terminal acidic products that inhibited the bioactivity of methanogens at a relatively low pH condition. The average biohydrogen production capacity increased with the NMWW proportion. The biohydrogen production rates in compartments 2–4 were 2.92, 10.17, and 12.85 L/day, respectively. The biohydrogen production in compartment 1 was significantly high, which was the most important fermentative biohydrogen production compartment in the ABR.

### 3.3. Soluble Fermentation Products

**Figure 8:**
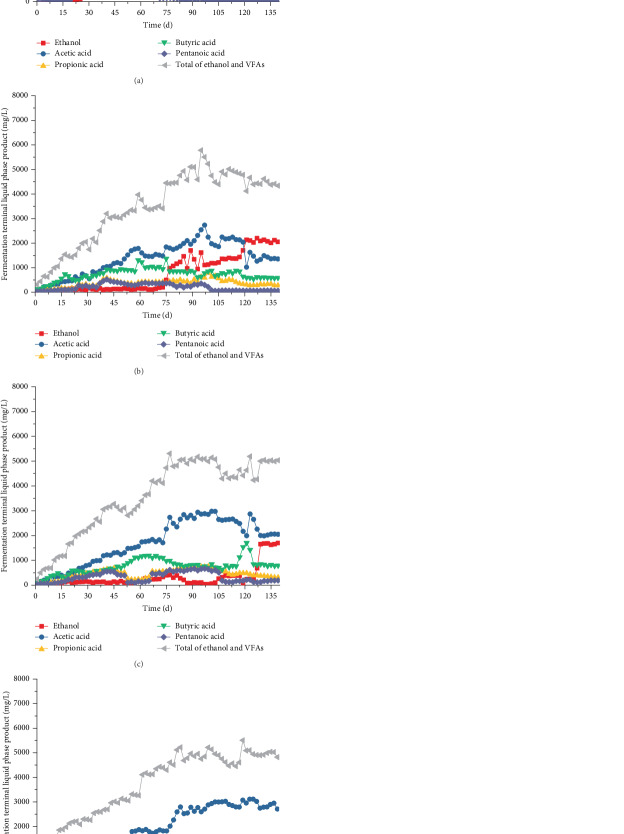
Variations in ethanol and VFAs ((a) compartment 1, (b) compartment 2, (c) compartment 3, and (d) compartment 4).

The variation in the soluble fermentation products showed a similar trend as the biogas production. At the initial period of each stage, the total VFAs and component oscillatory changed and then gradually stabilized. At the first stable state (days 64–73), the main soluble fermentation products in each compartment were acetic and butyric acid (Figure 8). The total acetic and butyric acid contents reached 62%, 72.8%, 73.6%, and 73.5% and presented butyric acid fermentation.

At the stable period of Stage II (days 108–117), the soluble fermentation products in compartment 1 were converted to ethanol and acetic acid, which accounted for 77.2% of the total VFAs and showed ethanol-type fermentation. The ethanol–acetic acid proportion in compartment 2 was 72.9% (Figure 8(b)), and the fermentation characteristic of AnAS exhibited ethanol-type fermentation. The acetic acid content in compartment 3 accounted for 59.5% of the total VFAs, and the ethanol, propionic acid, and butyric acid contents were 8.5%, 10.9%, and 18.2% (Figure 8(c)), respectively. This result indicated the establishment of mixed fermentation characteristics. The butyric acid content in compartment 4 was significantly higher than that of propionic acid, and the acetic and butyric acid contents accounted for 82.8% (Figure 8(d)) of the total VFAs, showing butyric acid-type fermentation.

Ethanol-type fermentation in compartment 1 was strengthened at the stable period of Stage III (days 130–139), and the ethanol–acetic acid proportion increased to 85.3%. Methane was not detected in the process. Thus, the methanogen activity in compartment 1 was effectively inhibited. Although compartment 2 was maintained in ethanol-type fermentation, the acetic acid content decreased from 44.7% to 31.3% (Figure 8(b)). This result suggests that methane production through acetic acid was enhanced. The ethanol content in compartment 3 increased to 33.2%, whereas the acetic and butyric acid contents decreased to 42.5% and 16%, respectively. The ethanol and acetic acid contents accounted for 75.7%, showing the ethanol-type fermentation property. In compartment 4, although the ethanol content increased significantly from 3.5% to 10.1% in Stage II, it was significantly lower than that of butyric acid. Therefore, the fermentation characteristic was still butyric acid-type fermentation.

With the increase in the NMWW proportion, the total VFAs of ABR showed an increasing trend. During the three stabilization stages, the average influent CODs were 5967, 6872, and 8084 mg/L, and the total effluent VFAs were 4264, 4578, and 4948 mg/L. The accumulation of the terminal acidic products decreased the pH in the ABR, which further inhibited the microbial activity of methanogens. The production and accumulation of ethanol and VFAs indicated that organic matter degradation in NMWW was incomplete, which may directly influence the removal efficiency of COD in the ABR.

### 3.4. ABR Operation Characteristics

#### 3.4.1. pH and Alkalinity

**Figure 9:**
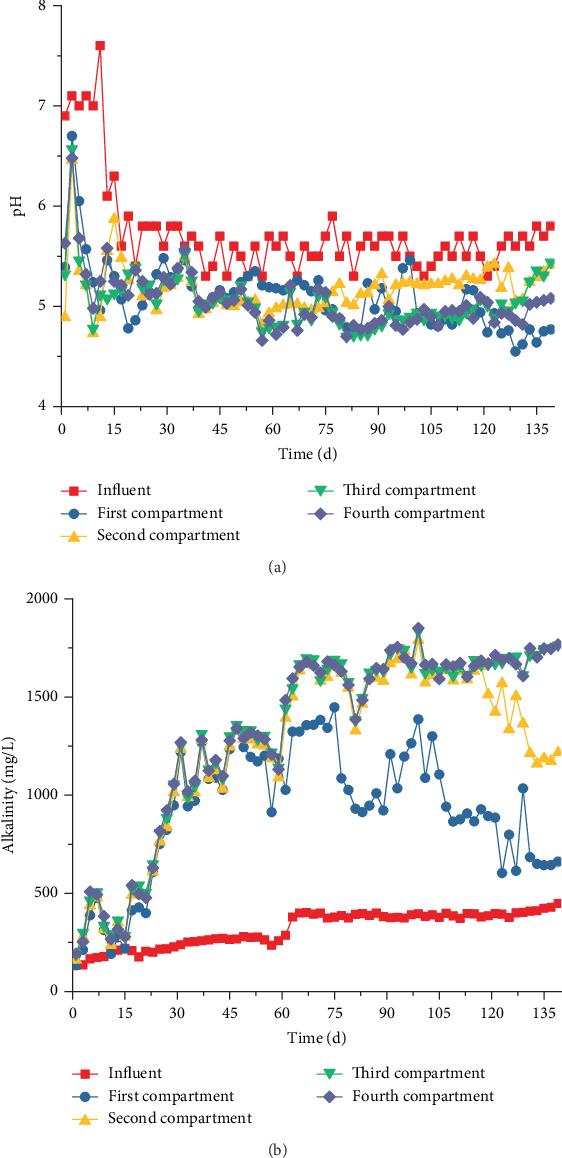
Effluent pH and alkalinity in each compartment ((a) pH; (b) alkalinity).

A considerable amount of VFA was produced due to AnAS fermentation, which resulted in relatively low pH values in the four compartments of ABR. The average pH and alkalinity were 5.2 and 1348 mg/L, respectively (Figure 9), and the pH value in compartment 1 was the highest. The alkalinity of each compartment also increased with the NMWW proportion. In Stage II (days 74–88), the pH value in compartment 2 increased, whereas the alkalinity in compartment 1 continuously decreased. From day 101 to 117, the pH value in compartment 2 was the highest, the pH variations in compartments 1 and 2 were similar, and the pH values in compartments 3 and 4 were stable (Figure 9(a)). With the increase in the NMWW proportion, the pH value in compartment 1 decreased rapidly to 4.6, and the pH value in compartment 2 was still the highest. From day 129, the pH value in each compartment began to increase, and the pH value in compartment 3 was close to that in compartment 2. In Stage III, the influent alkalinity gradually increased, and the alkalinity in compartments 1 and 2 decreased (Figure 9(b)).

The pH value in the ABR mainly depended on VFAs and alkalinity contents, and the variation in alkalinity was essentially based on the relative balance of CO_3_^2−^ and HCO_3_^−^ concentrations. When the pH value of the ABR was <5.0, the alkalinity was principally formed by HCO_3_^−^. The consumption of HCO_3_^−^ could decrease the alkalinity and pH when the residual VFAs increased. The HCO_3_^−^ content was highly correlated with the CO_2_ production in the ABR, and the produced CO_2_ concentration was small at the beginning of Stage I. Therefore, the relatively low HCO_3_^−^ decreased pH and alkalinity (Figure 9). However, the biogas production and VFA concentrations in each compartment increased rapidly after day 17, indicating that AnAS adapted to new circumstances; thus, additional CO_2_ was produced. With the increase in alkalinity, the buffering capacity of ABR was improved, and the pH values became increasingly stable. The pH value in compartment 1 was the lowest during the start-up process of ABR (days 0–27) due to the high VFA production and consumption of influent alkalinity. With the increase in the NMWW proportion, the alkalinity was accumulated; however, additional VFAs were produced, and the pH value in compartments 2–4 remained stable at 5.1.

#### 3.4.2. COD Removal

**Figure 10:**
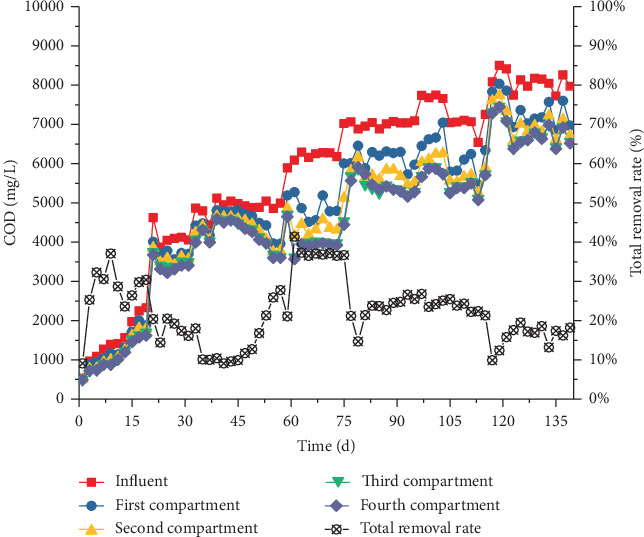
Variation and removal of COD in each compartment.

During the ABR operation in Stage I, the effluent COD in each compartment presented fluctuation property. Although effluent COD showed a decreasing trend, the discrepancy of COD in each compartment was small. On day 24, the influent COD was 4047 mg/L, and the effluent CODs in compartments 1–4 were 3780, 3586, 3388, and 3218 mg/L (Figure 10), respectively. The average COD removal rate was 15.1%. COD removal gradually increased and reached a stable level after day 43, and the average effluents in the four compartments were 5056, 4872, 4353, and 4192 mg/L, respectively. COD removal mainly occurred in compartments 1–3, and the least in compartment 4. In Stage II, the influent COD increased to approximately 7000 mg/L, and the COD removal rate rapidly decreased to 14.7%. With the ABR operation, the COD removal gradually increased to 24.0%. The influent COD increased to approximately 8000 mg/L in Stage III, and the COD removal rapidly decreased to 15.0%, whereas the COD removal rate gradually increased to 17.2% and remained stable after 123 days.

## 4. Discussions

### 4.1. Biohydrogen Production Activity and Fermentation Types

pH is an important ecological factor that affects the type of acid production, and the variation in pH values not only influences biohydrogen production but also leads to the variation in microbial community structure [19]. In the ABR biohydrogen production system, a large number of VFAs were produced due to the acidification and fermentation of microorganisms, which resulted in the low pH in each compartment, and the pH values changed in the range of 4.5–5.4. During the stable period of Stage I, the average pH and alkalinity values in compartment 1 were 5.2 and 1348 mg/L (Figure 9), respectively, and the metabolic characteristic of AnAS was butyric acid fermentation. The respective pH and alkalinity decreased to below 5.0 and 875 mg/L during the stabilization period of Stages II and III, and the fermentation type converted from butyric acid fermentation to ethanol-type fermentation. The pH and alkalinity in compartment 2 were significantly different. However, the fermentation type slightly changed because a large number of byproducts were introduced from compartment 1. The variation in metabolic characteristics in compartment 3 was overtly typical. With the increase in the NMWW proportion, the pH and alkalinity gradually increased from 5.0 and 1493 mg/L to 5.3 and 1785 mg/L (Figure 9). The metabolic characteristic transformed from butyric acid fermentation to mixed fermentation and finally formed ethanol fermentation. In compartment 4, given the positive influence of buffer effect in compartments 1–3, the variations in pH and alkalinity were slightly small (5.0 and 1514–1784 mg/L), and the metabolic characteristic maintained butyric acid fermentation.

COD removal was mainly through the methanogenesis of the methanogenic flora [20]. However, the ABR system was characterized by acidogenic fermentation flora and showed relatively low COD removal performance due to the bioactivity of methanogens [8]. COD could also be effectively removed by microbial synthesis and the release of CO_2_ and H_2_. Thus, additional VFAs remained and presented low COD removal efficiency. As shown in Figure 10, effluent COD decreased through each compartment; however, the total COD removal rates were only 37.6%, 21.4%, and 15.4% in the stable periods of Stages I–III, with corresponding biohydrogen production capacities by COD removal of 45.29, 241.04, and 360.22 L/kg COD, respectively. With the increase in the NMWW proportion (up to 80%), the biohydrogen production rate significantly improved (Figures 6 and 7). The soluble terminal products in the four compartments were dominated by acetic and butyric acid, and the average COD removal rate was 37.6%. The average biohydrogen production was 3.2 L/day, and the specific biohydrogen production by COD removal rate was 45.29 L/kg COD. When the influent NMWW proportion increased to 80% (COD of 8000 mg/L), the first three compartments showed ethanol-type fermentation, whereas butyric acid fermentation was strengthened in compartment 4. The average COD removal rate reduced to 15.4%, and the average biohydrogen and biohydrogen production capacity by COD removal increased to 12.85 L/day and 360.22 L/kg COD, respectively.

The initial biomasses (MLVSS) in compartments 1–4 were 6.9, 14.8, 23.1, and 22.7 g/L (Figure 2), respectively. With the ABR operation, the biomass in compartment 1 increased to 9.5 g/L at the first stable state in Stage I (day 66), whereas the biomass in compartments 2–4 decreased significantly to 12.9, 17.0, and 14.8 g/L, respectively. The biomass in compartment 1 increased to 11.5 g/L at the stable period of Stage II (day 112), whereas the biomass in compartments 2–4 continuously decreased. In Stage III, the biomass in compartments 2–4 were 8.0, 8.7, 11.0, and 9.3 g/L, respectively. The bioactivity of AnAS was further enhanced with high influent COD, and the biogas production significantly increased, which caused uplift and loss of AnAS in each compartment. The microbial activity of the AnAS in each compartment continuously improved with the increase in the NMWW proportion, and the specific biohydrogen production rates were 41.52, 123.55, and 217.5 L/kg MLVSS in Stages I–III, respectively.

### 4.2. Biohydrogen Production Efficiency in ABR

Although the biomass in compartments 2–4 gradually decreased, the biogas and biohydrogen production rates showed an upward trend. During the stable period of Stages I–III, the specific biogas production rates in compartment 2 were 116, 205, and 254 L/kg MLVSS·day, whereas those in compartment 3 were 101, 111, and 167 L/kg MLVSS·day and those in compartment 4 were 80, 194, and 232 L/kg MLVSS·day, respectively. The metabolic activity of AnAS in each compartment enhanced biohydrogen production efficiency. During the stabilization period of Stages I–III, the biohydrogen production rates in compartment 2 were 0.27, 0.39, and 2.38 L/day; those in compartment 3 were 0, 0.16, and 0.52 L/day; and the biohydrogen production rates for compartment 4 were 0.01, 0.01, and 0.02 L/day, respectively.

The variation in the biogas production rate in compartment 1 was significantly different from those in the subsequent three compartments. The biogas production rate reached the highest in the stable period of Stage II, which increased from 19.3 L/day to 35.4 L/day. However, the biogas production rate reduced to 31.2 L/day in Stage III. Although the biomass decreased, the specific biogas production rate increased, which were 282, 428, and 541 L/kg MLVSS·day, and the final biohydrogen production rates were 2.6, 9.6, and 10.0 L/day. Compartment 1 produced the most biohydrogen in the stable period of Stages I and II, and the contribution rate reached above 90%. However, in Stage III, the contribution rate decreased to 77.4% because the biohydrogen production efficiency in compartment II was significantly enhanced. Although a certain amount of hydrogen-producing acetogens was enriched in other compartments, their contribution to the total biohydrogen production of ABR was relatively low due to the limitation of fermentation substrates (carbohydrates) and hydrogen consumption of methanogens and homoacetogen [21]. Compartment 2 showed the highest contribution rate of 18.5% in Stage III, whereas compartment 4 presented little contribution rate for biohydrogen production.

### 4.3. Performance of Hydrogen-Producing Acetogen and Biohydrogen Production Efficiency

The methanogens were gradually eliminated by increasing influent COD and VFAs and reducing pH values to enrich the hydrogen-producing acetogens in the ABR. As shown in Figure 8, the acetic acid concentration increased with the decrease in ethanol and butyric acid. Thus, the performance of biohydrogen and acetogenic production was evident, and the hydrogen-producing acetogens were enriched well [22]. However, the average biohydrogen production rate was only 12.85 L/day under the optimum operation conditions, which was significantly lower than the result in the research conducted by van Ginkel [23].

When the influent COD gradually increased, the pH value reduced to lower than 5.2; however, the methanogenic activity was not completely inhibited in the ABR. The H_2_ and CO_2_ contents decreased with the increase in methane content in fermentation biogas (Figure 7). The amount of biohydrogen production was considerably affected due to the hydrogen consumption property of methanogens [24]. A relatively low hydrogen partial pressure was beneficial to the performance of hydrogen-producing acetogens, and the hydrogen-consuming homoacetogen and methanogens were generally the symbiotic bacteria with hydrogen-producing acetogens [25, 26]. Therefore, the existence of homoacetogen and methanogens was not conducive to the enrichment of hydrogen-producing acetogens and subsequently limited the biohydrogen production capacity in the ABR.

## 5. Conclusions

The biogas and biohydrogen production rates of ABR increased from 31.27 and 2.92 L/day to 61.54 and 12.85 L/day, respectively, when the influent COD increased to 8000 mg/L. Compartment 1 contributes most biohydrogen production, which accounted for >77.4%. When the influent COD was increased to 8000 mg/L, the pH of each compartment decreased continuously, and the fermentation metabolism characteristics significantly changed. The first three compartments eventually formed ethanol-type fermentation, whereas the butyric acid fermentation in compartment 4 was further enhanced. Although the biohydrogen production and acetogenic was improved in the ABR, the hydrogen-consuming bacteria methanogens and homoacetogen were effectively inhibited, which considerably affected the biohydrogen production efficiency. When the specific biogas production rate reached 232 L/kg MLVSS·day, the specific biohydrogen production rate was only 48 L/kg MLVSS·day.

## Figures and Tables

**Table 1:** Start-up parameters of ABR.

Operation phase	HRT (h)	*T* (°C)	Influent pH	Influent COD (mg/L)	NMWW proportion	Organic load rate (kg COD/m^3^·day)
Stage I (days 1–73)	24	35 ± 1	6.8–7.0	6131–6338	30%	0.54–6.11
Stage II (days 74–118)	24	35 ± 1	4.3–4.5	6868–7107	55%	6.56–7.66
Stage III (days 119–139)	24	35 ± 1	4.1–4.3	7718–8466	80%	7.73–8.49

## Data Availability

The data used to support the findings of this study are included within the article.
